# Migration of an Implantable Loop Recorder: A Meta-summary of Case Reports

**DOI:** 10.19102/icrm.2025.16056

**Published:** 2025-05-15

**Authors:** Alfredo Mauriello, Anna Rago, Dario Amore, Giacomo Sica, Antonello D’Andrea, Vincenzo Russo

**Affiliations:** 1 Cardiology Unit, Department of Medical and Translational Sciences, University of Campania “Luigi Vanvitelli,” Monaldi Hospital, Naples, Italy; 2 Cardiology and Intensive Care Unit, Department of Cardiology, Umberto I Hospital, Nocera Inferiore, Italy; 3 Thoracic Surgery Unit, Monaldi Hospital, Naples, Italy; 4 Department of Radiology, Monaldi Hospital, Naples, Italy

**Keywords:** Atrial fibrillation, implantable loop recorder, migration, remote monitoring, syncope

## Abstract

The migration of an implantable loop recorder (ILR) is a rare complication. We aimed to perform a meta-summary of case reports to characterize patients who experienced an ILR migration. We searched for case reports published in PubMed, Google Scholar, Scopus, and Embase from January 2017 to 2023 using the following keywords: “migration ILR,” “migration loop recorder,” “complication loop recorder,” and “complication ILR.” Seven case reports/case series reporting ILR migration were included. Data about patients’ characteristics, ILR implantation, time of onset, management, and clinical outcome of this complication were collected. Seven patients who experienced the migration of an ILR were examined. All patients experienced migration within 35 days following ILR implantation. The clinical suspicion of ILR migration mainly arose from patients’ symptomatology. The migration of the ILR was confirmed by a radiological scan in all cases, and surgical removal, preferably by video-assisted thoracic surgery, was required. In conclusion, intrapleural migration is a rare complication of ILR implantation. It may occur in the early postprocedural period. Clinical suspicion arises from symptoms, but a radiological scan is necessary to confirm the diagnosis. Surgical removal is mandatory.

## Introduction

An implantable loop recorder (ILR) is indicated in patients with unexplained syncope in whom a comprehensive non-invasive evaluation has failed to identify the cause of transient loss of consciousness.^1^ The standard ILR insertion position is a 45° angle relative to the sternum, or alternatively parallel to the sternum, over the fourth intercostal space.^2,3^ The most frequent complications are local pain and hematoma. To date, only a few cases of ILR migration have been reported.^2–8^ We aimed to complete a meta-summary of case reports to characterize patients who experienced an ILR migration by investigating comorbidities, time of onset, and diagnostic and interventional management of this complication.

## Materials and methods

**Figure 1:**
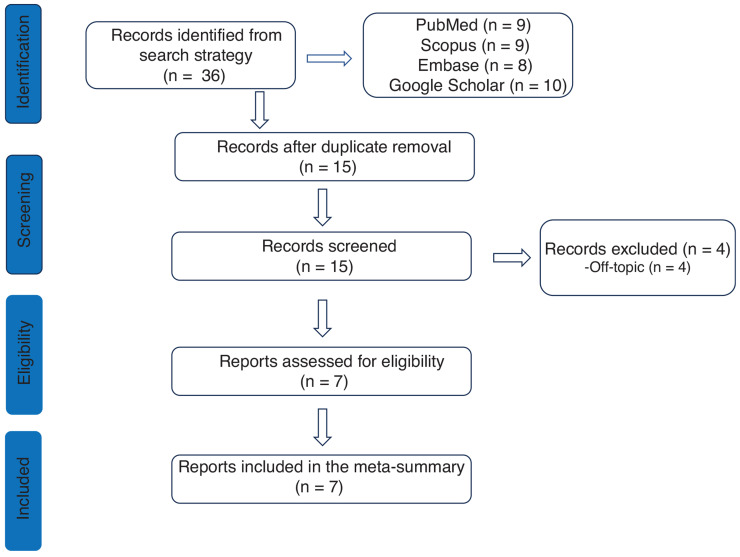
Flowchart of the selection process.

We searched for case reports published in PubMed, Google Scholar, Scopus, and Embase from January 2017 to 2023 using the following keywords: “migration ILR,” “migration loop recorder,” “complication loop recorder,” and “complication ILR.” Only English-language original reports were considered, and double-published papers were ruled out. Titles and abstracts were independently screened by two researchers (V.R. and A.M.), and discrepancies were resolved by discussion. Finally, six eligible case reports/case series were included in our meta-summary. The diagram of study selection is shown in **Figure 1**.

## Results

A total of seven patients who experienced the migration of an ILR were examined **(Table 1)**. The median age was 71 (interquartile range, 36–80) years, and 57.1% were women. The indications for an ILR implantation were recurrent unexplained syncope in six patients (85.7%) and arrhythmia detection in one patient (14.3%). The ILRs implanted were the Biomonitor II/III (Biotronik, Berlin, Germany) and Medtronic LINQ (Medtronic Inc., Minneapolis, MN, USA) in four (57%) and three (43%) patients, respectively. All ILRs were reportedly implanted using standard techniques, under local anesthesia. No data were available about the angle of ILR penetration or operators’ experience. Regarding the time to event, all patients experienced migration within 35 days following ILR implantation. No information about the possible causes of ILR migration was provided. An intraprocedural chest X-ray (CXR) was performed in two patients (33.3%) to check the correct positioning of the ILR. The clinical suspicion of ILR migration arose from patients’ symptomatology—in particular, local pain, discomfort in the migration area, and dyspnea in five cases (71%); loss of signal on ILR interrogation by the anterior chest in one case (14.5%); and unexpected R-wave amplitude flotation at ILR remote monitoring in one case (14.5%). The migration of the ILR was confirmed by CXR and computed tomography (CT) imaging in all cases. ILRs migrated most commonly into the left inferior part of the pleural cavity (57.14%), followed by the anterior–inferior part of the pleural cavity (14.28%), anterior mediastinum (14.28%), and superior abdomen (14.28%). ILR migration was managed by device removal through video-assisted thoracic surgery (VATS) (n = 5; 71.4%) or open surgical intervention (n = 2; 28.6%).

**Table 1:** Characteristics, Diagnosis, and Management of Patients with Implantable Loop Recorder Migration

Year	Authors	Age (Years)	Sex	Comorbidities	ILR Indication	ILR Type	Time to Migration (Days)	Diagnosis	Migration	Surgical Removal
2017	Preminger et al.^2^	78	Male	Hypertension, dyslipidemia, sleep apnea	AF	Medtronic LINQ	35	CXR and CT	Left anteroinferior pleural cavity	VATS
2019	Hasnie et al.^3^	36	Male	—	Unexplained syncope	Medtronic LINQ	5	CXR and chest–abdomen CT	Superior abdomen	Open
2021	Rahkovich and Laish-Farkash^4^	65	Female	—	Recurrent syncope	Biomonitor III	7	CXR and CT	Left posteroinferior pleural cavity	VATS
2021	Basyal et al.^5^	71	Female	—	Recurrent syncope	Biomonitor III	—	CXR and CT	Anterior mediastinum	Open
2022	Ang et al.^6^	58	Female	—	Syncope by SSS	Medtronic LINQ	28	CXR and CT	Left anteroinferior pleural cavity	VATS
2023	Signore et al.^7^	80	Female	Hypertension, diabetes	Unexplained syncope	Biomonitor III	7	CXR and CT	Left posteroinferior pleural cavity	VATS
2024	Russo et al.^8^	75	Male	Hypertension, dyslipidemia, COPD, CKD	T-LOC	Biomonitor III	1	CXR and CT	Anterior costophrenic recess	VATS

*Abbreviations:* AF, atrial fibrillation; CKD, chronic kidney disease; COPD, chronic obstructive pulmonary disease; CT, computerized tomography; CXR, chest X-ray; ILR, implantable loop recorder; SSS, sick sinus syndrome; T-LOC, transient loss of consciousness; VATS, video-assisted thoracic surgery.

## Discussion

The diagnosis and treatment of intrathoracic foreign bodies was first reported in surviving World War II veterans.^9^ Initially, the main indications for removing intrathoracic metallic foreign bodies were the patient’s symptomatology and localization in the posterior mediastinum and diaphragm; over time, the removal of foreign bodies, even if asymptomatic, was considered mandatory due to the high risk of dangerous complications (hemoptysis, bronchiectasis, lung abscesses).^10,11^ Recently, some cases of intrathoracic migration of medical devices, such as Kirschner wires, have been reported^12,13^; thoracoscopy is considered the preferred approach.^12^ The migration mechanism remained unclear; however, it might include muscular activity, shoulder movements, breathing movements, negative intrathoracic pressure with respiratory excursion, and gravitational force.^12^

ILR implantation is an easy and feasible procedure characterized by low intraoperative complications. Short-term local pain in the insertion site and hematomas are the most frequent complications; rarely, ILR extrusion due to skin erosion occurs.^14^

ILR migration is a rare complication described in seven cases. In half of them, it occurred in the early post-implantation period (<7 days) and always within the following 35 days; therefore, it should be considered an early complication of ILR implantation. The relationship between ILR migration and the implantation technique has not been studied yet; however, it may be caused by an intraoperative technical mistake. In particular, when an excessive angle of penetration of >40° is applied, the pocket tool might be inadvertently inserted through the intercostal space until the pleural cavity; moreover, if the tip of the device was initially implanted deep with an angulation toward the intercostal muscle, the thin chest wall structure and the negative pressure of the pleural cavity could determine the intrathoracic migration.

**Figure 2:**
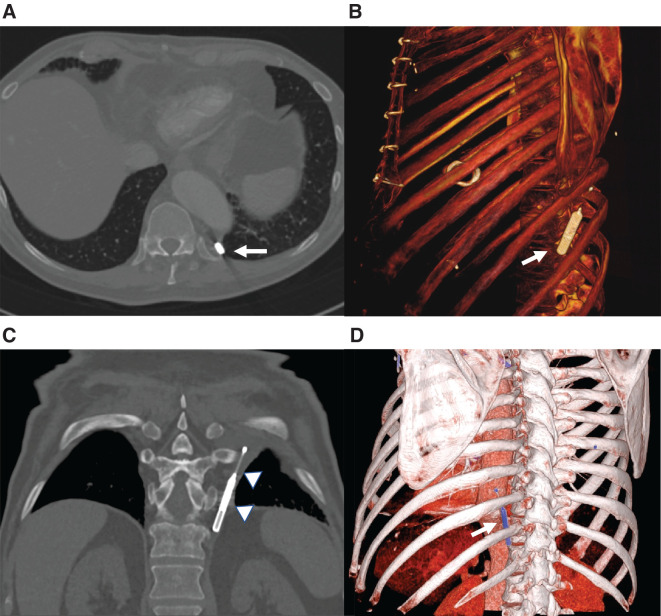
Computed tomography scan images confirming migration of the implantable loop recorder (ILR) into the left pleural cavity. **A:** The axial computed tomography scan shows the ILR in the posterior pleural cavity between the descending thoracic aorta and the costal skeleton (arrow). **B**, **D**: Three-dimensional volume-rendered images with color mapping provide a realistic anatomical representation of the migrated ILR (arrows) useful for surgical planning. **C:** Coronal oblique multiplanar reformation shows the ILR with its coil in the pleural cavity (arrowheads). No pleural effusion was present.

The suspicion of an ILR migration is mainly driven by the patient’s symptomatology, such as local pain, discomfort in the migration area, and dyspnea; however, the increasing use of ILR remote monitoring may speed up the diagnosis by the early detection of sudden and unexpected R-wave amplitude flotation.^15^ CXR and CT scans are useful to confirm the diagnosis of ILR migration **(Figure 2)**. ILR removal should be preferably performed by VATS.

### Limitations

The low number of included cases is certainly a limitation; however, the present study is the first meta-summary regarding this topic. The specific implant approaches performed in the various reports could not be verified. The generation of ILR devices and the experience of implantation centers were different across case reports. The implantation procedures, preventive measures, and timing of follow-up were not standardized, limiting further analysis.

## Conclusion

Intrapleural migration is a rare, but not negligible, complication of ILR implantation. It may occur in the early postprocedural period, generally within 7 days and always within the following 35 days. The clinical suspicion arises from symptoms such as pain, discomfort, and dyspnea. A CT scan is necessary to confirm the diagnosis. Surgical removal, preferably by VATS, is mandatory. Larger studies are needed to increase the knowledge and improve the prevention of ILR migration.
